# Preparation of konjac oligoglucomannans with different molecular weights and their *in vitro* and *in vivo* antioxidant activities

**DOI:** 10.1515/biol-2020-0076

**Published:** 2020-11-11

**Authors:** Weidong Yang

**Affiliations:** College of Chemistry and Chemical Engineering, Baoji University of Arts and Sciences, Baoji, 721013, China

**Keywords:** konjac oligoglucomannan (KOGM), molecular weight, antioxidant activity, orthogonal test

## Abstract

In this paper, konjac oligoglucomannan (KOGM) was obtained with a hydrolysis rate of 56.24% by controlling the hydrolysis conditions. KOGM was passed through a 0.2 kDa dialysis bag, a 3 kDa ultrafiltration tube, and a 5 kDa ultrafiltration tube, creating samples with molecular weights of 0.2–3 kDa (IV), 3–5 kDa (III), and >5 kDa (II), respectively. The *in vitro* antioxidant activities of the KOGM samples were tested by measuring their removal effects on ˙OH, {\text{O}}_{2}^{-}, and DPPH˙. The *in vivo* antioxidant activities of the samples were analyzed by measuring their impacts on the malondialdehyde (MDA) content, superoxide dismutase (SOD) activity, and glutathione peroxidase (GSH-PX) activity in mice. The results show that the KOGM samples in groups III and IV could effectively remove ˙OH, {\text{O}}_{2}^{-}, and DPPH˙; the KOGM samples in all three groups could enhance the SOD and GSH-PX activities and reduce the MDA content in the liver tissues of mice; finally, the antioxidant activity of KOGM is negatively correlated with the molecular weight.

## 1 Introduction

Konjac is a perennial herbaceous plant belonging to the genus *Amorphophallus*, Araceae family. Its main active component is konjac glucomannan (KGM), a water-soluble natural polysaccharide [1]. KGM is generally considered to be formed by linking glucose and mannose in a ratio of 1:1.69 or 1.4:1 through β-1,4 glycosidic bonds; at the C3 position of the mannose group in the main chain, there are branched chains connected by β-1,3 bonds. With a molecular weight of 200–20,000 kDa, KGM has an acetyl group at the C6 position for every 19 sugar units in the main chain [2,3].

The application of KGM is bottlenecked by its large molecular weight, high viscosity, and low solubility. Therefore, much attention has been paid to the degradation of KGM into konjac oligoglucomannan (KOGM), a functional oligosaccharide consisting of 2–10 monosaccharide units. KOGM can be obtained by degradation through enzymatic hydrolysis, chemical method, and physical method [4]. Studies have shown that KOGM can enhance immunity, lower blood lipids and blood sugar, and slow down the aging process [5,6,7,8,9,10].

Recently, it has been learned that KOGMs with different molecular weights differ in structure and biological effect. Ma et al. [11] demonstrated that the increasing molecular weight of KOGM pushes up many physico–chemical properties: corn starch solubility, pseudoplasticity, viscoelasticity, gelatinization temperature, gelatinization time, and aging inhibition. Peng et al. [12] explored the gel properties of KOGMs with different molecular weights and proved that the gel properties of KOGM are best at a molecular weight of 650–700 kDa. Gao et al. [13] examined the sulfonated KOGMs with different molecular weights and revealed that KOGM with a molecular weight of 10–30 kDa has the best anticoagulant, antitumor, and antibacterial effects. Li et al. [14] found that KOGM has an optimal hypoglycemic effect when its molecular weight falls within a range of 30–80 kDa. However, there are very few reports on the oxidation performance of KOGMs with different molecular weights. It is meaningful to prepare KOGMs with different molecular weights and to explore their resistance to oxidation.

In this paper, konjac gum is hydrolyzed with β-mannanase. By controlling the hydrolysis conditions, KOGM samples were obtained with a hydrolysis rate of 56.24%. Then, the KOGM samples with different molecular weights were separated through dialysis and centrifugal ultrafiltration. The composition, *in vivo* antioxidant activity [15,16,17,18], and *in vitro* antioxidant activity of these samples were evaluated in detail.

## 2 Materials and methods

### 2.1 Materials

KGM (purity: 95%) was purchased from Wuhan Qingjiang Konjac Products Co., Ltd. β-Mannanase (activity ≥3,145 U/g) was purchased from Mianyang Habio Bioengineering Co., Ltd. Mice (50% males, 50% females; weight: 20 ± 2 g) were purchased from Shaanxi Medical Laboratory Animal Center. Malondialdehyde (MDA), superoxide dismutase (SOD), and glutathione peroxidase (GSH-PX) detection kits were purchased from Shanghai Jianglai Biotechnology Co., Ltd. The dialysis bags were purchased from Shanghai Qiaoxing Trading Co., Ltd. The other reagents were all of analytical grade and produced domestically.

### 2.2 Methods

#### 2.2.1 Preparation of KOGMs with different molecular weights

First, 5 g of konjac powder was evenly dispersed into 100 mL of 0.2 mol/L acetate buffer to obtain a 5% (w/v) solution. The solution was mixed thoroughly with β-mannanase (dosage: 30–150 U/g) to initiate the hydrolysis. The mixture reacted for 1–5 h in a water bath at pH of 3.0–7.0 and temperature of 30–70°C. After that, the mixture was taken out and boiled for 10 min to inactivate the enzyme and stop the hydrolysis. Then, the reaction solution was centrifuged at 4,000 rpm to remove the unreacted KGM. The supernatant was taken for a 0.2 h dialysis in a 0.2 kDa dialysis bag to remove monosaccharides and small molecule salts from the sample, creating sample KOGM I. Next, KOGM I was passed through a 5 kDa ultrafiltration tube, producing sample KOGM II with a molecular weight greater than 5 kDa and a dialysate with a molecular weight of less than 5 kDa. The dialysate was further filtered through a 3 kDa ultrafiltration tube, producing sample KOGM III with a molecular weight of 3–5 kDa and sample KOGM IV with a molecular weight of less than 3 kDa. The samples were freeze-dried for further use.

#### 2.2.2 Determination of KGM hydrolysis rate

The content of reducing sugar was determined by the dinitrosalicylic acid colorimetric method [19]. The KGM hydrolysis rate was calculated using the equation (1):

(1) \text{Hydrolysis rate}\hspace{.5em}( \% )=\frac{{M}_{1}-{M}_{2}}{M}\times 100,

where *M*_1_ and *M*_2_ are the total contents of reducing sugar before and after hydrolysis, respectively, and *M* is the content of the KGM.

#### 2.2.3 Orthogonal test

To achieve the best degradation effect, it is important to optimize parameters for enzymatic degradation. Assuming all other factors as constants, the commonly used single-factor test examines only one factor, without considering the interaction between parameters. Hence, this test approach cannot judge whether multiple parameters are optimal. As a result, the orthogonal test was selected to determine the optimal parameters.

According to the orthogonal test design, the KGM hydrolysis rate was taken as the index to be evaluated. Then, four parameters of enzymatic hydrolysis were optimized, including time (A), temperature (B), pH (C), and enzyme dosage (D). Each factor was tested at three levels (Table 1). The results were subjected to orthogonal analysis to optimize the preparation conditions for the KOGM.

**Table 1:** Factors and levels of the orthogonal test

Parameter	Level		
	1	2	3
(A) Time (h)	1	2	3
(B) Temperature (°C)	45	50	55
(C) pH	4.5	5	5.5
(D) Enzyme dosage (U/g)	50	90	130

#### 2.2.4 Determination of number-average molecular weight (*M*_n_) of KOGM [20]

The *M*_n_ of KOGM was measured by quantifying the hydrolysate composition through high-performance liquid chromatography. The parameters were configured as follows: chromatographic column, Shodex NH_2_P-50 4E (4.6 mm × 250 mm); mobile phase, water:acetonitrile = 65:35; flow rate, 1 mL/min; column temperature, 35°C; injection volume, 15 µL; and detector, refractive index detector.

#### 2.2.5 Determination of *in vitro* antioxidant activity

##### 2.2.5.1 ˙OH removal capacity [21]

The ˙OH removal capacity of KOGM was tested by the Fenton’s reagent method. First, 150 µL of the KOGM samples with different contents was placed in separate test tubes. Then, 300 µL of 1.8 mmol/L ferrous sulfate solution, 200 µL of 1.8 mmol/L sodium salicylate–ethanol solution, and 25 µL of 0.03% hydrogen peroxide were added in turn into each test tube. The mixture in each tube was shaken well and reacted for 30 min at 37°C. The absorbance of each solution was measured at the wavelength of 510 nm. Vitamin C (Vc) and distilled water were selected as the positive control and the blank control, respectively. The ˙OH removal rate was calculated using the equation (2):

(2) \text{Removal}\hspace{.5em}\text{rate}\hspace{.5em}( \% )=\frac{{A}_{0}-({A}_{1}-{A}_{2})}{{A}_{0}}\times 100,

where *A*_0_, *A*_1_, and *A*_2_ are the absorbances of the blank control, the sample groups and positive control, and 200 µL of ferrous sulfate solution as the reagent blank.

##### 2.2.5.2 DPPH˙ removal capacity [8]

First, 0.2 mL of KOGM with different hydrolysis rates was mixed with 4 mL of 120 µmol/L DPPH˙ solution in separate test tubes. The mixture in each test tube was shaken well and placed in darkness for 30 min. Taking absolute ethanol as the blank, the absorbance of the solution in each test tube was measured at 548 nm. Vc and distilled water were selected as the positive control and the blank control, respectively. The DPPH˙ removal rate was calculated using the equation (2), where *A*_2_ is changed to the absorbance measured by replacing the DPPH˙ solution with a 95% ethanol solution as the reagent blank.

##### {\text{O}}_{2}^{-} removal capacity [22]

2.2.5.3

The removal effect of KOGM on {\text{O}}_{2}^{-} was determined by the pyrogallol method. First, 0.5 mL of KOGM with different hydrolysis rates was mixed evenly with 5 mL of 50 mmol/L Tris–HCl buffer (pH: 8.2) and preheated for 10 min in a water bath at 37°C. Then, 1.0 mL of 3.5 mmol/L pyrogallol solution was added. After 6 min of reaction, 0.5 mL of 8 mmol/L HCl was added quickly to terminate the reaction. The absorbance of each solution was measured at the wavelength of 420 nm. Vc and distilled water were selected as the positive control and the blank control, respectively. The removal rate of {\text{O}}_{2}^{-} was calculated using the equation (3):

(3) \text{Removal}\hspace{.5em}\text{rate}\hspace{.5em}( \% )=\frac{{A}_{0}-{A}_{1}}{{A}_{0}}\times 100,

where *A*_0_ and *A*_1_ are the absorbances of the blank control and the sample groups and positive control, respectively.

##### 2.2.6 Determination of *in vivo* antioxidant activity [23]

Fifty days before the experiment, 50 mice were fed basic diet for 5 days and were randomly divided into a normal control group (A), three KOGM experimental groups (B1, B2, and B3), and a positive control group (C). In each group, the number of male mice was equal to the number of female mice.

The normal control group was fed with 0.25 mL of normal saline every day; the three KOGM groups were fed with 80 mg/kg (i.e., 0.25 mL 6.4 mg/mL) KOGM II, KOGM III, and KOGM IV, respectively, every day. The positive control group was fed with 50 mg/kg Vc every day.

The mice were free to eat and drink water in the 2-week-long experiment. In the end, the mice were fasted for 12 h. The eyeballs were removed for blood collection, using heparin as an anticoagulant. The mice were killed through cervical dislocation. Then, the livers were taken out and made into 10% liver tissue homogenate. Finally, the SOD activity, GSH-PX activity, and MDA content were measured.


**Ethical approval:** The research related to animal use has been complied with all the relevant national regulations and institutional policies for the care and use of animals.

##### 2.2.7 Statistical analysis

All experiments were performed in triplicate (*n* = 3) unless otherwise specified. Except that the orthogonal experiment was expressed by the mean, the others were expressed as mean ± standard deviation (SD). A one-way analysis of variance (ANOVA) was performed using SPSS version 19, and Duncan’s multiple range test was performed to determine the statistical differences among different samples. Differences at *P* < 0.05 were considered significant, and *P* < 0.01 was considered extremely significant.

## 3 Results

### 3.1 Results of single-factor tests

#### 3.1.1 Effect of time on KGM hydrolysis rate

The effect of time on the hydrolysis rate was tested at a temperature of 50°C, pH 5, and an enzyme dosage of 90 U/g.

**Figure 1:**
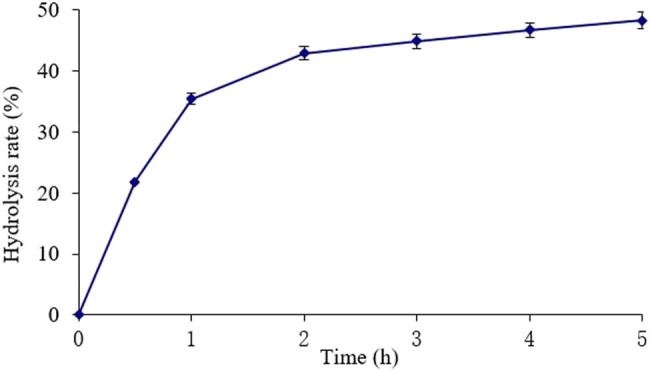
Effect of time on KGM hydrolysis rate.

As shown in Figure 1, the hydrolysis rate gradually increased with time. The hydrolysis increased rapidly before 2 h, especially in the first 0.5 h of the reaction (*P* < 0.01). After 2 h, the hydrolysis rate increased slowly (*P* < 0.05). The trend basically conforms to the kinetic features of the enzymatic reaction. In the beginning, the high concentration of substrate promotes the reaction in the direction of product formation; with the lapse of time, the hydrolysis rate stabilizes due to enzyme inactivation and product inhibition [24].

#### 3.1.2 Effect of temperature on KGM hydrolysis rate

The effect of temperature on the hydrolysis rate was tested at time 2 h, pH 5, and an enzyme dosage of 90 U/g.

**Figure 2:**
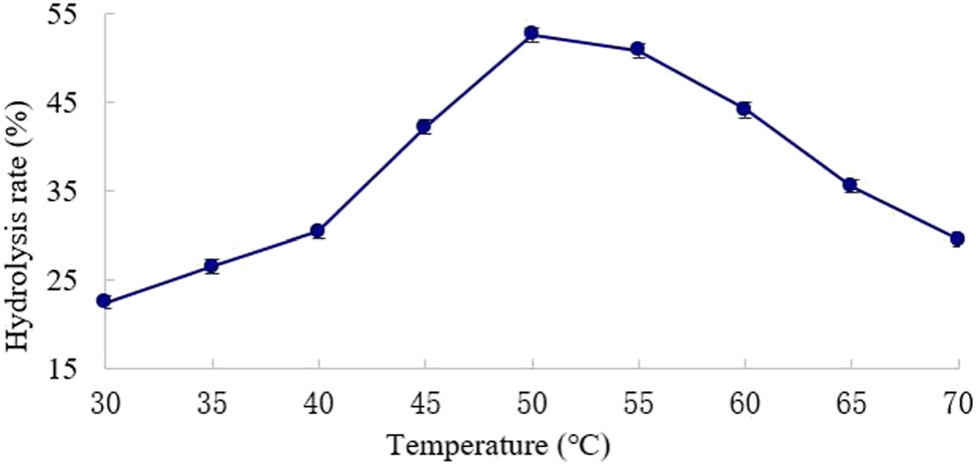
Effect of temperature on KGM hydrolysis rate.

As shown in Figure 2, the hydrolysis rate increased (*P* < 0.05) with temperature before reaching 50°C. This is because the enzymatic hydrolysis of konjac gum requires a certain activation energy. Meanwhile, high temperature makes konjac gum more soluble and KGM less viscous, thereby promoting the mass transfer of the reaction system. The hydrolysis rate peaked at 50°C. However, the enzyme became less stable and active at higher temperatures, causing a reduction in the hydrolysis rate.

#### 3.1.3 Effect of pH on KGM hydrolysis rate

The effect of pH on the hydrolysis rate was tested at a time of 2 h, a temperature of 50°C, and an enzyme dosage of 90 U/g.

**Figure 3:**
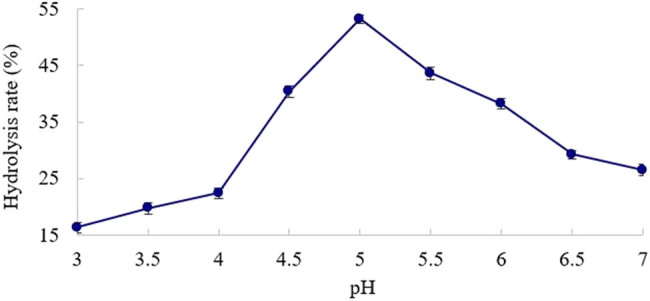
Effect of pH on KGM hydrolysis rate.

As shown in Figure 3, β-mannanase exhibited a relatively good catalytic effect under acidic conditions. The optimal pH for enzymatic hydrolysis is 5.0. When the pH was below or above 5.0, the catalytic activity of the enzyme was greatly reduced. The main reason is that the excessively high or low pH affects the dissociation state of the active groups of the enzyme, reducing the chance for the enzyme to bind to the substrate [25].

#### 3.1.4 Effect of enzyme dosage on KGM hydrolysis rate

The effect of enzyme dosage on the hydrolysis rate was tested at a time of 2 h, a temperature of 50°C, and pH 5.

**Figure 4:**
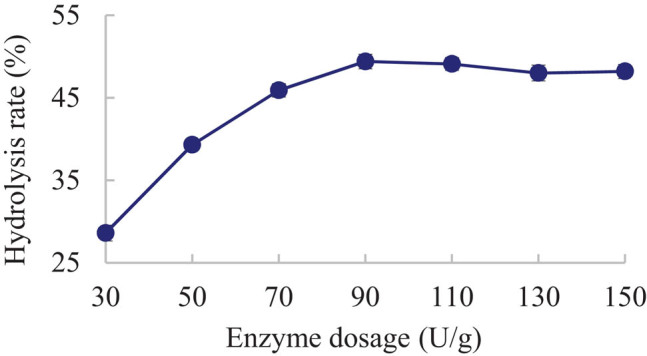
Effect of enzyme dosage on KGM hydrolysis rate.

As shown in Figure 4, the hydrolysis rate initially increased with the enzyme dosage (*P* < 0.05), because the enzymatic reaction is insufficient at the low enzyme dosage. The hydrolysis rate reached the maximum when the enzyme dosage reached 90 U/g substrate. The hydrolysis rate did not increase, despite a further increase in enzyme dosage; there was no more substrate left for the extra enzyme molecules to bind with.

### 3.2 Results of the orthogonal test

The effect of each parameter on the KGM hydrolysis rate was revealed by the above results of single-factor tests. On this basis, time (A), temperature (B), pH (C), and enzyme dosage (D) were selected for an L_9_(3^4^) orthogonal test of four factors and three levels. The results of the orthogonal test are listed in Table 2, where *K* is the sum of the levels under each factor and *R* = (*K*_max_ − *K*_min_)/3 is the influence range of each factor.

**Table 2:** Results of the orthogonal test

Experimental no	(A) Time (h)	(B) Temperature (°C)	(C) pH	(D) Enzyme dosage (U/g)	Hydrolysis rate (%)
1	1	45	4.5	50	45.87
2	1	50	5	90	53.45
3	1	55	5.5	130	49.36
4	2	45	5	130	56.48
5	2	50	5.5	50	49.71
6	2	55	4.5	90	42.87
7	3	45	5.5	90	39.16
8	3	50	4.5	130	43.09
9	3	55	5	50	31.02
K_1_	148.68	141.51	131.83	126.60	
K_2_	149.06	146.25	140.95	135.48	
K_3_	113.27	123.25	138.23	148.93	
R	11.93	7.67	3.04	7.44	

Based on the *R* values in Table 2, the four factors were ranked as A > B > D > C in descending order of the effect on hydrolysis rate. According to ANOVA, the optimal parameter combination was A_2_B_2_C_2_D_3_: a time of 2 h, a temperature of 50°C, pH 5, and an enzyme dosage of 130 U/g.

### 3.3 *M*_n_ values of KOGMs with different molecular weights

Under the optimal parameter combination, the hydrolysis rate of konjac powder was 56.24%. After enzymolysis, each konjac powder solution was subjected to ultrafiltration and then *M*_n_ was measured (Table 3). The result (*M*_n_ = 1,328) shows that the molecular weight of konjac powder decreased after enzymolysis. The *M*_n_ of KOGM IV, which was obtained through 3 kDa centrifugal ultrafiltration, was 773. Hence, KOGM samples with different molecular weights can be acquired efficiently and quickly from KGM through β-mannanase hydrolysis and ultrafiltration.

**Table 3:** Measured *M*_n_ values of the samples

Sample	*M*_n_
KGM	64,530
KOGM I	1,328
KOGM II	1,352
KOGM III	1,176
KOGM IV	773

### 3.4 *In vitro* antioxidant activities of KOGMs with different molecular weights

#### 3.4.1 ˙OH removal capacity

**Figure 5:**
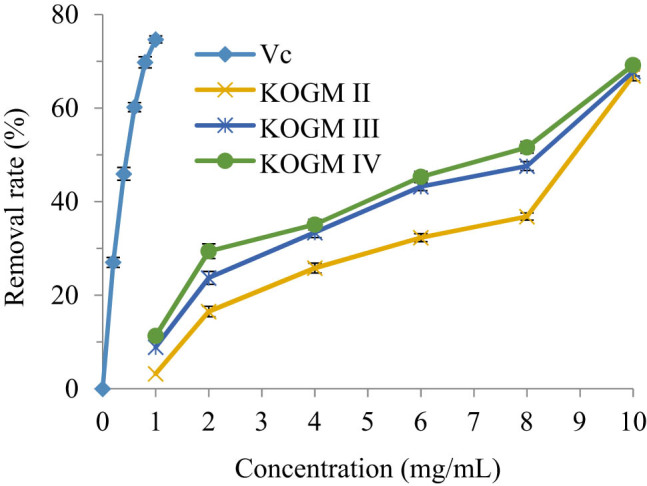
˙OH removal effects of the KOGMs with different molecular weights and Vc.

The ˙OH, as the most active radical, causes serious damage to adjacent biomolecules [26]. As shown in Figure 5, when their concentrations increased within 0–10 mg/mL, the ˙OH removal effects of the KOGMs with different molecular weights and Vc were on the rise. The strongest ˙OH removal effect belongs to KOGM IV (molecular weight: 200–3,000 Da); the ˙OH removal effect of 10 mg/mL KOGM IV was comparable to that of 1 mg/mL Vc. The weakest ˙OH removal effect belongs to KOGM II. The smaller the molecular weight, the better the ˙OH removal effects of the KOGM. The test results of KOGM IV were fitted by the least squares (LS) method, producing a one-variable quadratic polynomial: *y* = 0.3554*x* ^2^ + 0.5407*x* + 27.24 (*R*² = 0.987). Thus, the IC_50_ of KOGM IV for the ˙OH was 7.2779 mg/mL.

#### 3.4.2 DPPH˙ removal capacity

**Figure 6:**
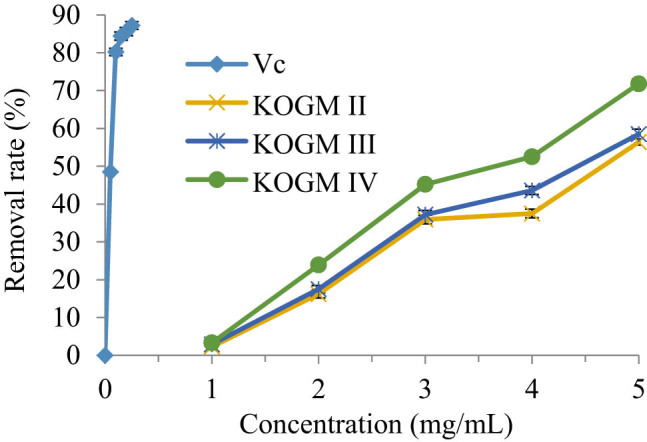
DPPH˙ removal effects of the KOGMs with different molecular weights and Vc.

DPPH˙ is a stable lipophilic free radical, which has been widely used to evaluate the antioxidant activity of food and pharmaceutical raw materials [27]. As shown in Figure 6, when their concentrations increased within 0–5 mg/mL, the DPPH˙ removal effects of the KOGMs with different molecular weights and Vc were on the rise. The smaller the molecular weight, the better the removal effect. However, DPPH˙ was not removed at a low KOGM concentration (<1 mg/mL), whereas 87.2% of the DPPH˙ were removed when the Vc concentration was 0.25 mg/mL. Hence, KOGM showed a much weaker DPPH˙ removal effect than Vc at low concentrations. By contrast, 5 mg/mL KOGM IV (molecular weight: 200–3,000 Da) removed 71.8% of the DPPH˙ about 82.34% of the removal effect of the Vc. Therefore, KOGM has a good removal effect of DPPH˙, especially at low molecular weight. The test results of KOGM IV were fitted by the LS method, producing a one-variable quadratic polynomial: *y* = −1.1857*x* ^2^ + 23.674*x* − 18.64 (*R*² = 0.987). Thus, the IC_50_ of KOGM IV for the DPPH˙ was 3.5199 mg/mL.

#### {\text{O}}_{2}^{-} removal capacity

3.4.3

**Figure 7:**
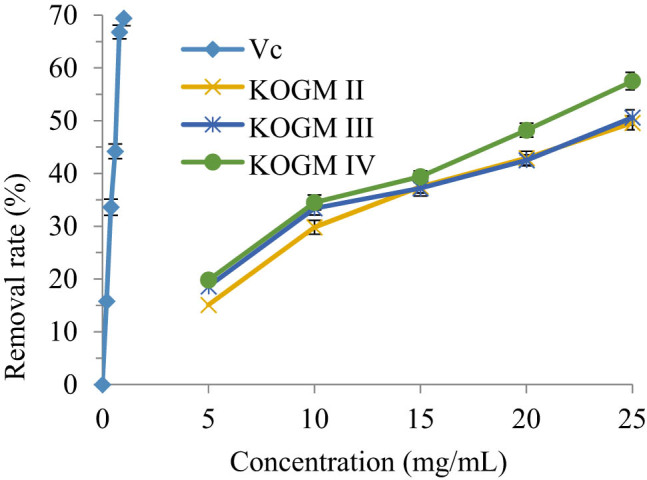
{\text{O}}_{2}^{-} removal effects of the KOGMs with different molecular weights and Vc.

Despite its inactive nature, {\text{O}}_{2}^{-} can peroxidize nucleic acids, unsaturated fatty acids, and proteins through disproportionation and oxidation reactions. The resulting free radicals pose a serious threat to the body [28]. As shown in Figure 7, when their concentrations increased within 0–25 mg/mL, the {\text{O}}_{2}^{-} removal effects of the KOGMs with different molecular weights and Vc were on the rise. The smaller the molecular weight, the better the removal effect. The removal effect of KOGM was poorer than the Vc, as the latter could remove 69.4% of the {\text{O}}_{2}^{-} at a concentration of 0.25 mg/mL. However, 25 mg/mL KOGM IV (molecular weight: 200–3,000 Da) removed 57.5% of the {\text{O}}_{2}^{-}, about 82.85% of the removal effect of the Vc. Therefore, KOGM has a good removal effect of {\text{O}}_{2}^{-}, especially at low molecular weight. The test results of KOGM IV were fitted by the LS method, producing a one-variable quadratic polynomial: *y* = −0.0544*x* ^2^ + 3.5166*x* + 1.7321 (*R*² = 0.9852). Thus, the IC_50_ of KOGM IV for the {\text{O}}_{2}^{-} was 19.7753 mg/mL.

### 3.5 *In vivo* antioxidant activities of KOGMs with different molecular weights

The SOD and GSH-PX are the most important antioxidant enzymes in the body. They can remove free radicals on time and protect the body from oxidative damage. The MDA is a lipid peroxide produced under the imbalance between the oxidation system and antioxidant system of the body. The growth in the MDA will damage the cells. The MDA has often been used to measure the lipid peroxidation damage [29].

As shown in Table 4, the KOGM groups and positive control group were much higher than the normal control group in terms of the SOD and GSH-PX activities in the liver tissues, and lower than the latter in terms of the MDA content. Compared with the normal group, the KOGM IV group and the positive control group were the only two groups with very low MDA contents in the liver tissues; the KOGM III group and the positive control group were very high in terms of the SOD activity in the liver tissues, whereas KOGM IV had an extremely high SOD activity, about 1.22 times than that of the positive control group; the KOGM IV group and the positive control group were extremely high in terms of the GSH-PX activity in the liver tissues, whereas the other KOGM groups were very high in that respect.

**Table 4:** Influence of molecular weight of KOGM on the MDA, SOD, and GSH-PX levels in the liver tissues of mice (\bar{x}\pm s,\hspace{.5em}n=10)

Groups	MDA (nmol mg prot^−1^)	SOD (U mg prot^−1^)	GSH-PX (U mg prot^−1^)
Normal group (A)	21.45 ± 2.61	53.67 ± 12.89	152.45 ± 14.67
KOGM II group (B1)	20.82 ± 3.13	51.98 ± 11.17	238.98 ± 25.49*
KOGM III group (B2)	19.13 ± 2.87	66.59 ± 12.49*	256.32 ± 18.02*
KOGM IV group (B3)	18.26 ± 3.39*	70.98 ± 12.56**	349.22 ± 35.08**
Positive control group (C)	16.59 ± 3.11**	58.16 ± 14.51*	336.96 ± 25.56**

Note: * *P* < 0.05 and ** *P* < 0.01 compared to the normal group.

## 4 Discussion

Although there have been many reports about the preparation of KOGM by degradation of KGM, most of them are studies on the degradation process by chemical, physical, and biological methods. There are few studies on the controllable degradation of KGM and the preparation of KOGMs with different molecular weights, and the molecular weight has a great impact on the efficiency of KOGM. In this paper, KGM was hydrolyzed by β-mannanase to prepare KOGM, and the best preparation process was obtained through the study of various factors of enzymatic hydrolysis. At the same time, through the orthogonal experimental analysis of various factors, it was found that the time of enzymatic hydrolysis had a significant impact on the enzymatic hydrolysis, so the controllable degradation of KGM could be realized by controlling the hydrolysis time. At the same time, three kinds of KOGMs with different molecular weights were obtained by centrifugation, dialysis, and ultrafiltration, which is the basis for the follow-up study of the biological function of KOGMs with different molecular weights and further exploration of the preparation of KOGMs with high oxidation resistance.

The metabolism of the human body produces a large number of free radicals. The free radicals of the body are in the dynamic balance of continuous generation and elimination. Once the free radicals in the body are produced too much or the body’s clearance ability is insufficient, the balance will be destroyed, which will cause adverse reactions such as aging and cancer [30]. In this study, three kinds of KOGMs with different molecular weights were tested for antioxidation *in vitro* and *in vivo*. *In vitro* experiments showed that KOGM had the ability to scavenge free radicals, and its scavenging ability was positively correlated with the concentration and negatively correlated with the molecular weight of KOGM. This is because the high concentration and low molecular weight of KOGM molecular chain contain more reduced hemiacetal hydroxyl, which can make the highly oxidized hydroxyl free reduction [31]; it has strong hydrogen supply capacity and can remove DPPH˙ with proton free radical; in addition, it contains high content of reducing ketone, which plays an antioxidant role by donating hydrogen atoms to destroy the free radical chain [32].

In human body, SOD can reduce {\text{O}}_{2}^{-} to H_2_O_2_ and O_2_, GSH-PX can reduce organic peroxide and H_2_O_2_ to H_2_O and nontoxic substances. If SOD and GSH-PX were present, the content of {\text{O}}_{2}^{-}, organic peroxide, and H_2_O_2_ would all decrease [33]. In this way, peroxidation damage of cells and mitochondria can be prevented, and the MDA content of lipid peroxide can be reduced. Experiments showed that KOGM could significantly enhance the activities of SOD and GSH-PX in the liver, and the activities increased significantly with the decrease of molecular weight, which provided a certain experimental basis for the antioxidant effect of KOGM. The increase of the activity of antioxidant enzymes in the liver of mice may be due to the activation of SOD and GSH-PX through the metabolic pathway *in vivo*, so that the activity of SOD and GSH-PX can be increased, and then the content of MDA can be reduced.

This study showed that KGM can be degraded in a controlled way and KOGMs with different molecular weights can be prepared by the enzymatic method. The antioxidative effect of KOGM was verified by experiments *in vitro* and *in vivo*, and the relationship between antioxidant and molecular weight was obtained, which lays a foundation for the preparation of high antioxidant KOGM and edible health products in future.

## 5 Conclusion and prospect

In this paper, the KOGM samples were prepared by hydrolyzing konjac powder for 2 h at a temperature of 50°C, pH 5, and a β-mannanase dosage of 130 U/g. The samples were subjected to ultrafiltration, creating three kinds of KOGMs with different molecular weights. Through *in vitro* and *in vivo* tests, KOGM was proved to have certain antioxidant activity, which was negatively correlated with the molecular weight; the antioxidant activity of KOGM was weaker than that of the Vc. Our research shows that the enzymatic method can successfully prepare the KOGMs with different molecular weights and that KOGM with a low molecular weight is a natural antioxidant.

However, the composition of KOGMs with a low molecular weight is still very complex and requires further study in order to elucidate a specific antioxidant mechanism. At the same time, it is not enough to verify the efficacy only in mice, and the use of the relevant knowledge and equipment in the field of clinical medicine is needed for further study to verify its antioxidant effect.
